# An Exploratory Study on Transcriptomic Profiling of Circulating miRNA Associated with Hub Genes in the Development of Polycystic Ovary Syndrome

**DOI:** 10.3390/ncrna12040030

**Published:** 2026-08-05

**Authors:** Yogesh Vetriselvan, Jayakumar Swetha, Manoranjani Murugan, Irisappan Ganesh, Vishnu Bhat Ballambattu, Pushpa Premanath Kotur, Deepa Shanmugam, Marcella Sherin Samuel, Sambandam Ravikumar

**Affiliations:** 1 Department of Medical Biotechnology, Faculty of Interdisciplinary Studies, Aarupadai Veedu Medical College and Hospital, Vinayaka Mission’s Research Foundation (DU), Kirumampakkam, Puducherry 607403, India; yogeshvetriselvan@avmc.edu.in (Y.V.); manoranjanimurugan@avmc.edu.in (M.M.); ganesh.irisappan@avmc.edu.in (I.G.); 2 Department of Biochemistry, Aarupadai Veedu Medical College and Hospital, Vinayaka Mission’s Research Foundation (DU), Kirumampakkam, Puducherry 607403, India; swetha.jayakumar@avmc.edu.in; 3 Central Research Laboratory, Vinayaka Mission’s Sankarachariyar Dental College, Salem 636308, India; 4 Medical Research and Publications, Aarupadai Veedu Medical College and Hospital, Vinayaka Mission’s Research Foundation (DU), Kirumampakkam, Puducherry 607403, India; drvishnubhat@yahoo.com; 5 Department of Obstetrics and Gynaecology, Aarupadai Veedu Medical College and Hospital, Vinayaka Mission’s Research Foundation (DU), Kirumampakkam, Puducherry 607403, India; pushpapremnathkotur@avmc.edu.in (P.P.K.); deepa.shanmugam@avmc.edu.in (D.S.); 6 Sandbox Innovation Fund Program, Massachusetts Institute of Technology, iHQ (Bldg E38), 292 Main St, Cambridge, MA 02142, USA; marce845@mit.edu

**Keywords:** polycystic ovary syndrome, microRNA, small RNA sequencing, differential expression

## Abstract

**Background:** Polycystic ovary syndrome (PCOS) is a complex endocrine disorder that affects women of reproductive age, often associated with metabolic issues, causing infertility. Although it is common, the cause of PCOS remains unknown, and early diagnosis is challenging. MicroRNAs (miRNAs) serve as post-transcriptional regulators of gene expression, play a significant role in PCOS development, and have emerged as biomarkers for reproductive and metabolic disorders. However, research on miRNA signatures in the Indian population is limited. This pilot exploratory study aims to compare the candidate differentially expressed miRNAs (DE miRNAs) in serum samples from individuals with and without PCOS using high-throughput miRNA sequencing. **Methods:** In the study, patients with PCOS and age-matched controls were included; small RNAs were isolated from their serum, libraries were prepared, and the libraries were analyzed by next-generation sequencing. Bioinformatics analysis, including miRBase annotation, differential expression analysis, target prediction, GO/KEGG enrichment, and hub gene network analysis, was performed. **Results:** A total of 967 miRNAs were identified, with seven showing differential expression (log2FC > 1, *p* < 0.05). Among these, six miRNAs of hsa-miR-219a-2-3p, hsa-mir-384, hsa-miR-149-5p, hsa-miR-3182, hsa-miR-3960, and hsa-miR-4508 were upregulated, whereas hsa-mir-139 was downregulated. Functional enrichment and hub gene analyses identified key targets, including *TP53*, *FOXO1*, *HIF1A*, and *HDAC1*, that are crucial to the cell cycle, insulin signaling, and hypoxia. **Conclusions:** These preliminary findings identify candidate serum miRNA signatures in Indian women that may be associated with PCOS’s reproductive and metabolic issues. These pilot study results suggest potential miRNAs and their target pathway links with PCOS that need validation in larger cohorts for diagnostic or therapeutic applications.

## 1. Introduction

Polycystic ovary syndrome (PCOS) is the most common endocrine and metabolic disorder affecting 13–20% of reproductive-aged women worldwide [1,2]. It is characterized by hyperandrogenism, chronic anovulation, and polycystic ovary morphology based on Rotterdam criteria [3]. This syndrome is often associated with infertility, which affects 70–80% of those with this syndrome, and is also associated with metabolic complications such as obesity, cardiovascular disorder, hypertension, as well as insulin resistance [4]. Although its etiology remains unclear, environmental, genetic, and epigenetic modifications contribute to the pathogenesis of this syndrome [5]. However, early diagnosis and monitoring of disease progression remain clinically challenging. Therefore, understanding the molecular mechanisms underlying the syndrome’s pathophysiology is needed and may lead to the identification of novel diagnostic and prognostic therapeutic targets for PCOS.

In recent years, microRNAs (miRNAs) have emerged as critical regulators in the pathogenesis of human diseases [6]. miRNAs are small, non-coding RNA molecules, typically 19–25 nucleotides long, that regulate gene expression post-transcriptionally by binding to target mRNAs, causing either mRNA degradation or translational repression [7]. They are involved in various cellular processes, including proliferation, differentiation, apoptosis, metabolism, and organogenesis [8]. Dysregulation of miRNA expression is linked to diseases like cancer, endocrine disorders, cardiovascular disorders, and metabolic conditions such as diabetes and obesity [9]. In PCOS, miRNA expression changes have been observed across various biological sources, including whole blood, follicular fluid, granulosa cells, and serum exosomes [10].

MiRNAs play a major role in regulating ovarian function, inflammation, and metabolic homeostasis in PCOS, and their altered expression suggests their potential need for diagnostic and prognostic biomarkers [11]. Most of the research shows that dysregulated miRNA expression contributes significantly to the development of PCOS, influencing ovarian function, follicular growth, steroid hormone production, and inflammatory processes [12]. To elucidate the molecular landscape of PCOS, miRNA sequencing was used to identify differentially expressed miRNAs, providing insights into miRNA-mediated regulatory pathways involved in PCOS [13].

However, several studies focus on specific candidate miRNAs, which often fail to capture the miRNA expression landscape and complex regulatory networks involved in PCOS [14]. High-throughput NGS technologies, such as miRNA sequencing, enable comprehensive profiling of the entire transcriptome, provide miRNAs with differential expression patterns, and reveal population-specific molecular signatures [5]. Numerous studies have examined miRNA expression using RT-PCR and its role in PCOS across Western and Chinese populations. In India, research on miRNA sequencing in PCOS remains limited, creating a gap in understanding the unique molecular signatures of PCOS in this demographic.

This exploratory study aimed to compare the expression of candidate differentially expressed miRNAs in serum samples from PCOS cases and aged-matched controls using miRNA sequencing, and further the identified miRNAs were validated by Real Time Polymerase Chain Reaction (RT-PCR) to investigate the potential of serum miRNAs as candidate biomarkers for metabolic and reproductive traits in PCOS among Indian women. These findings will further elucidate the molecular mechanisms underlying PCOS pathogenesis and contribute to its early detection and effective management.

## 2. Results

### 2.1. miRNA Read Count Quantification

**Figure 1:**
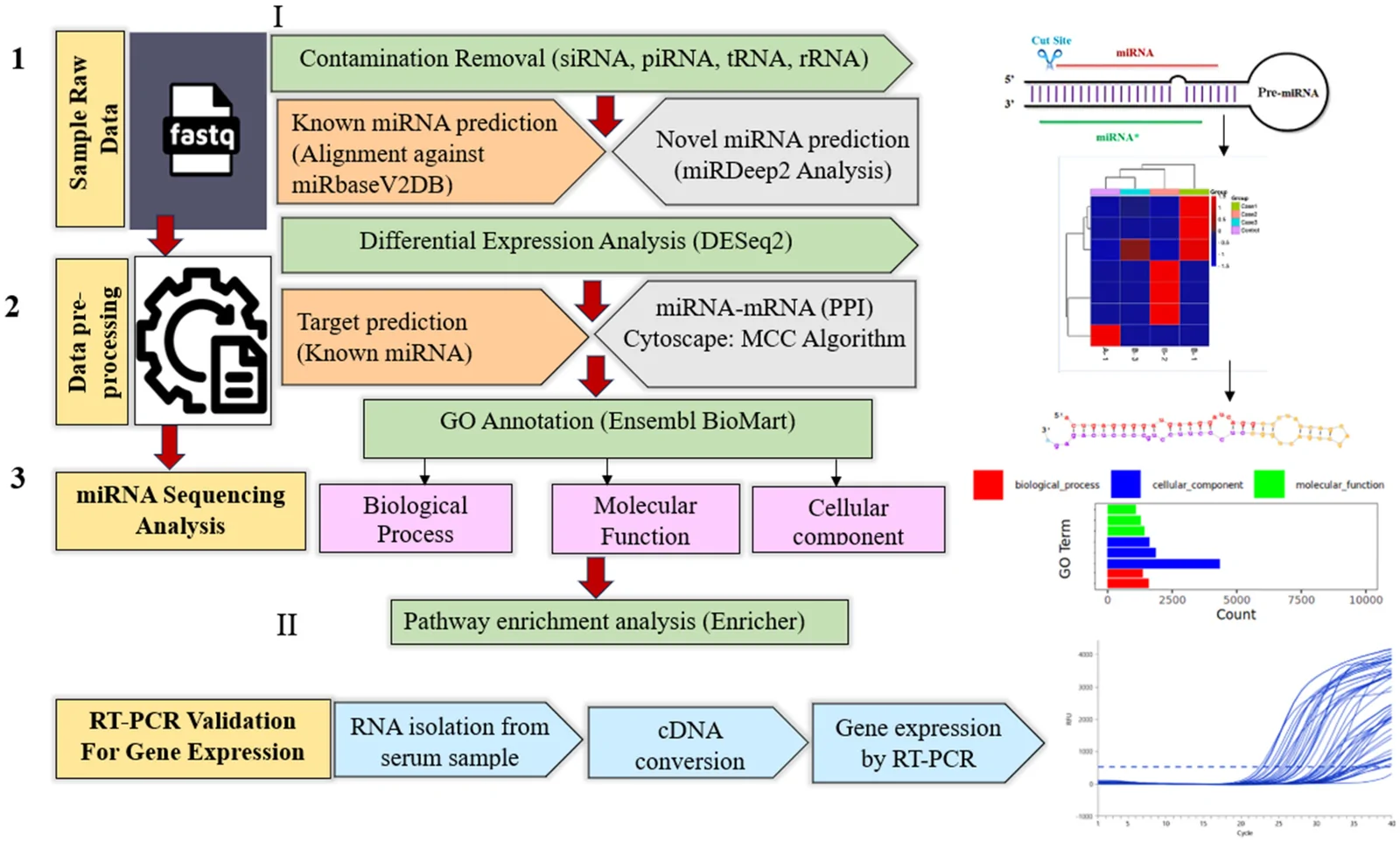
Overview of the small RNA sequencing workflow and bioinformatics analysis pipeline showing the various packages, software, and tools used in the study.

**Figure 2:**
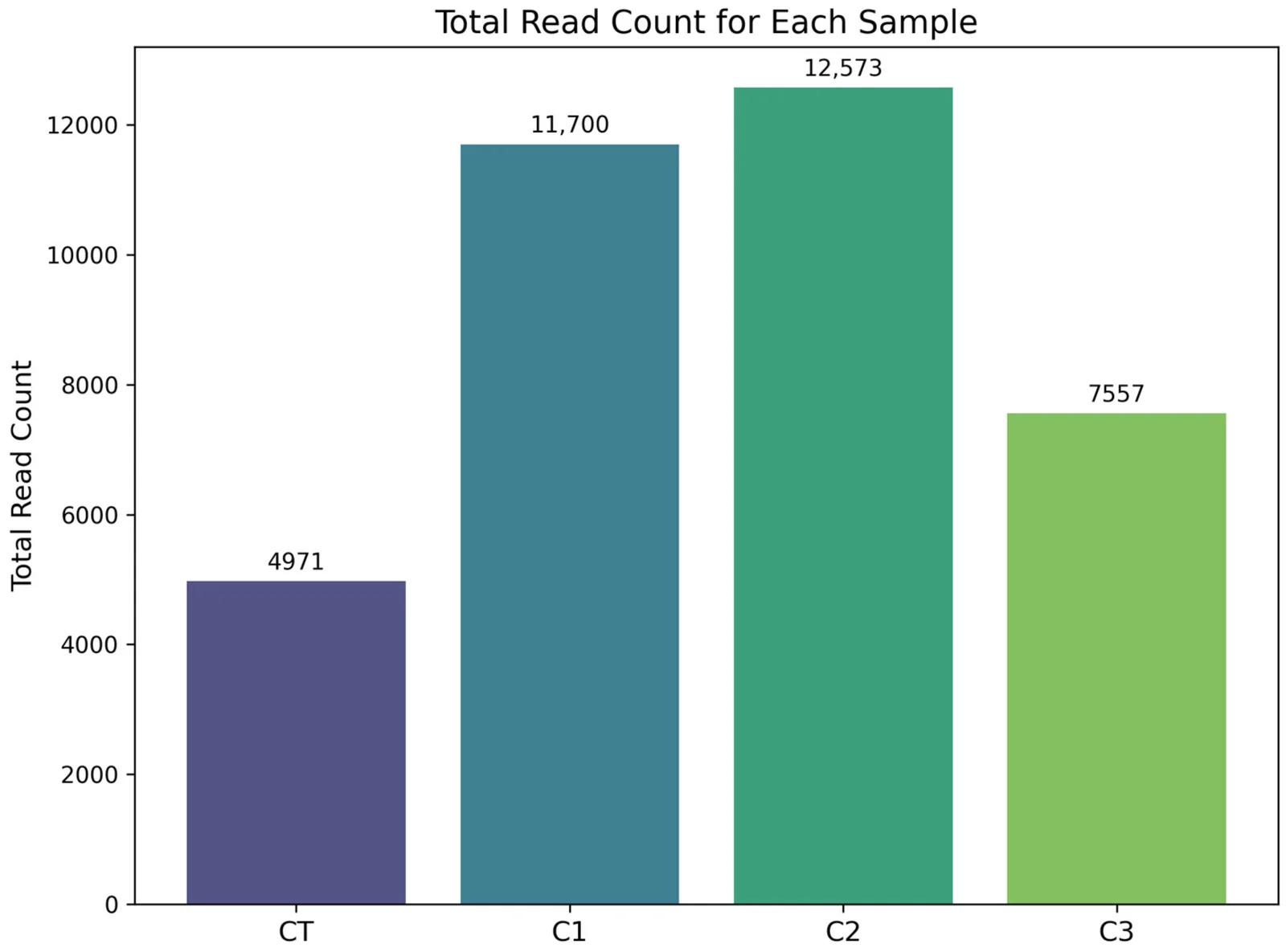
Bar graph displaying the total mapped miRNA read counts from small RNA sequencing in both PCOS cases and control samples, after aligning reads to the miRBase reference database.

Expression profiling of the genomics libraries from case and control samples, using their raw sequence reads, was mapped to the miRBase database to identify miRNAs using the size factor. The overview of the miRNA sequencing analysis for the study is shown in Figure 1. Based on the sequencing, the raw sequencing reads were identified as 20,491,480 for control, 19,856,573 for Case 1, 41,956,862 for Case 2, and 20,980,388 for Case 3. The unique miRNAs were identified as 367 for control, 566 for Case 1, 647 for Case 2, and 476 for Case 3. The count matrix quantified expression levels for MI: precursor miRNAs (mir) and MIMAT: mature miRNAs (miR). The total read count for each sample generated during the count matrix is shown in Figure 2. The raw miRNA counts from each sample were then processed using DESeq2 to normalize the data and identify statistically significant differential expression. In addition, QC metrics for input reads, trimmed reads, miRNA mapping, alignment efficiency, and the percentage of known miRNA reads, as well as canonical miRNAs and their corresponding isomiRs, are shown in Supplementary Table S1. Hemolysis assessment was based on read counts for hsa-miR-451a (MI0001729) and hsa-miR-23a (MI0000079), extracted from the known miRNA count matrix. hsa-miR-451a was detected in all four samples, whereas hsa-miR-23a consistently showed low read counts (0–2 reads).

### 2.2. Candidate Differentially Expressed miRNAs

**Figure 3:**
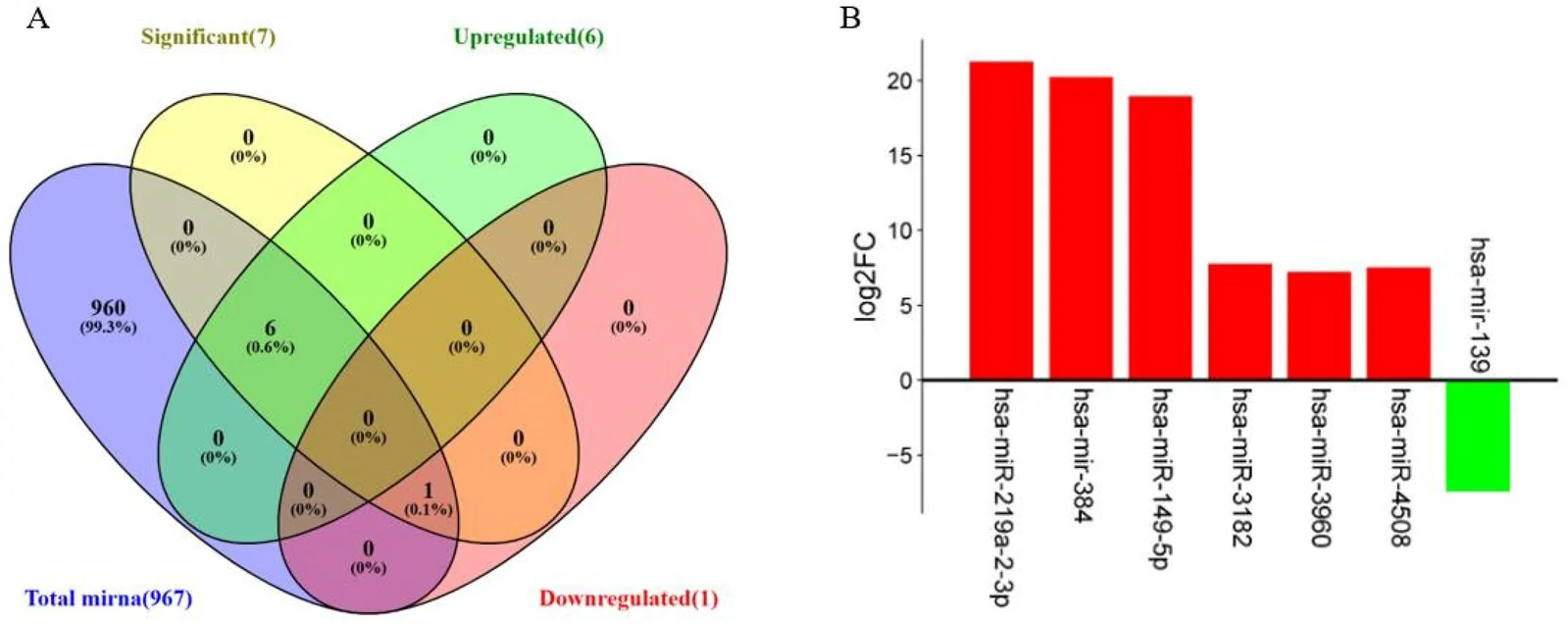
(**A**) Venn diagram showing the overlap and distribution of significantly differentially expressed miRNAs between PCOS cases and controls. Seven candidate miRNAs were identified as significantly dysregulated, based on log2Fold change > 1 and *p*-value < 0.05. (**B**) Bar plot showing the log2 fold change values of the seven significantly differentially expressed miRNAs identified in PCOS compared to controls. Upregulated miRNAs are displayed with positive fold change values, while downregulated miRNAs are shown with negative values.

**Figure 4:**
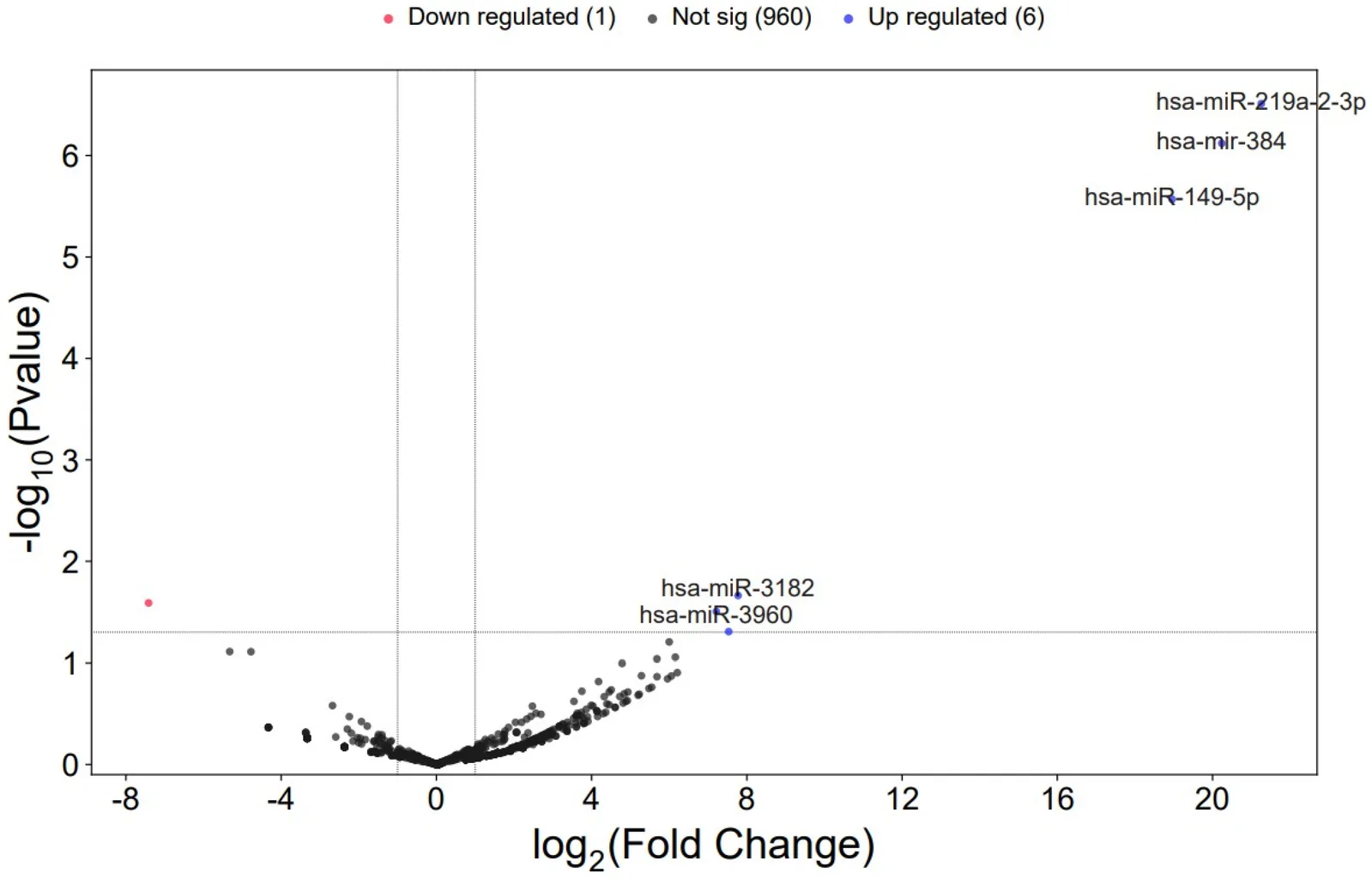
Volcano plot showing the results of miRNA differential expression analysis between the PCOS and control groups. The x-axis shows log2 fold change, while the y-axis displays −log10 (*p*-value). Significantly upregulated miRNAs are marked in blue, and downregulated ones in red. The significance thresholds used were log2FC > 1 and *p*-value < 0.05.

**Figure 5:**
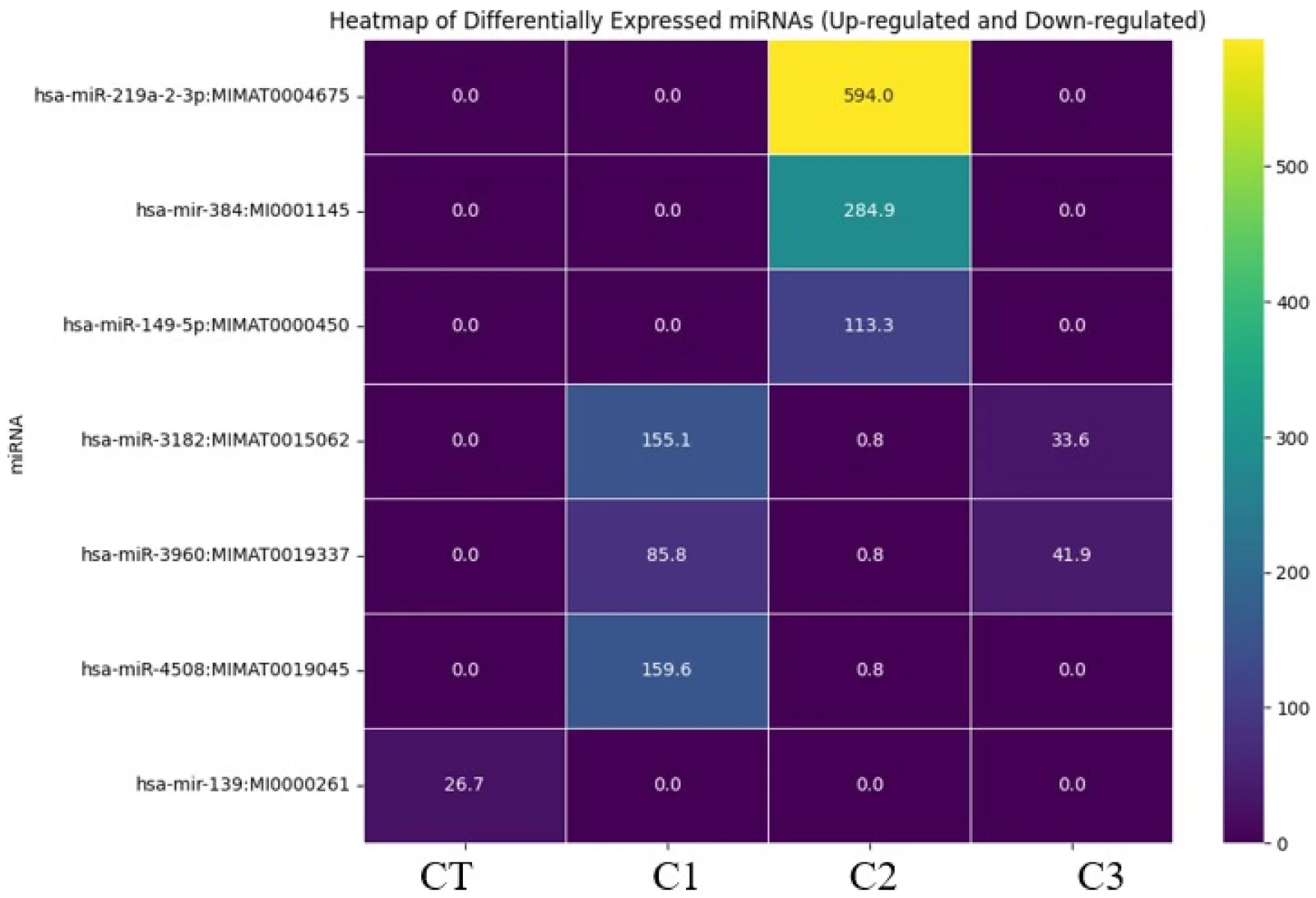
The heatmap shows dysregulated miRNA expression in PCOS and control samples. Values are normalized with DESeq2. Dark purple indicates low expression, yellow/green high expression. Clustering reveals distinct profiles.

Differential expression analysis was performed using the DESeq2 R package, which identified several miRNAs that are highly dysregulated in PCOS cases compared with controls, as shown in the Venn diagram in Figure 3A and the bar diagram in Figure 3B. A total of 967 miRNAs were identified in the samples, as shown in Supplemental Table S2. Of these, 306 (31.6%) met the minimum threshold of ≥5 reads, while the remaining 661 (68.4%) were categorized as low-confidence reads. Among them, seven candidate miRNAs showed differential expression, based on nominal statistical significance, fold change criteria and false discovery rate (FDR) correction, including hsa-miR-219a-2-3p (MIMAT0004675), hsa-mir-384 (MI0001145), hsa-miR-149-5p (MIMAT0000450), hsa-miR-3182 (MIMAT0015062), hsa-miR-3960 (MIMAT0019337), hsa-miR-4508 (MIMAT0019045), which were upregulated, and hsa-mir-139 (MI0000261), which was downregulated. These results are derived from base mean, log2Fold change, *p*-value, and adjusted *p*-value, as shown in Table 1. The identified miRNAs are shown in a volcano plot in Figure 4, where blue dots indicate upregulated miRNAs and red dots indicate downregulated miRNAs. In Figure 5, the heatmap shows the DE miRNA levels between the two groups. The control samples are indicated by low expression in dark purple, while high expression is shown in yellow to green, as shown in the heatmaps for hsa-miR-219a-2-3p, hsa-mir-384, and hsa-miR-149-5p.

### 2.3. Target mRNA Annotation

**Figure 6:**
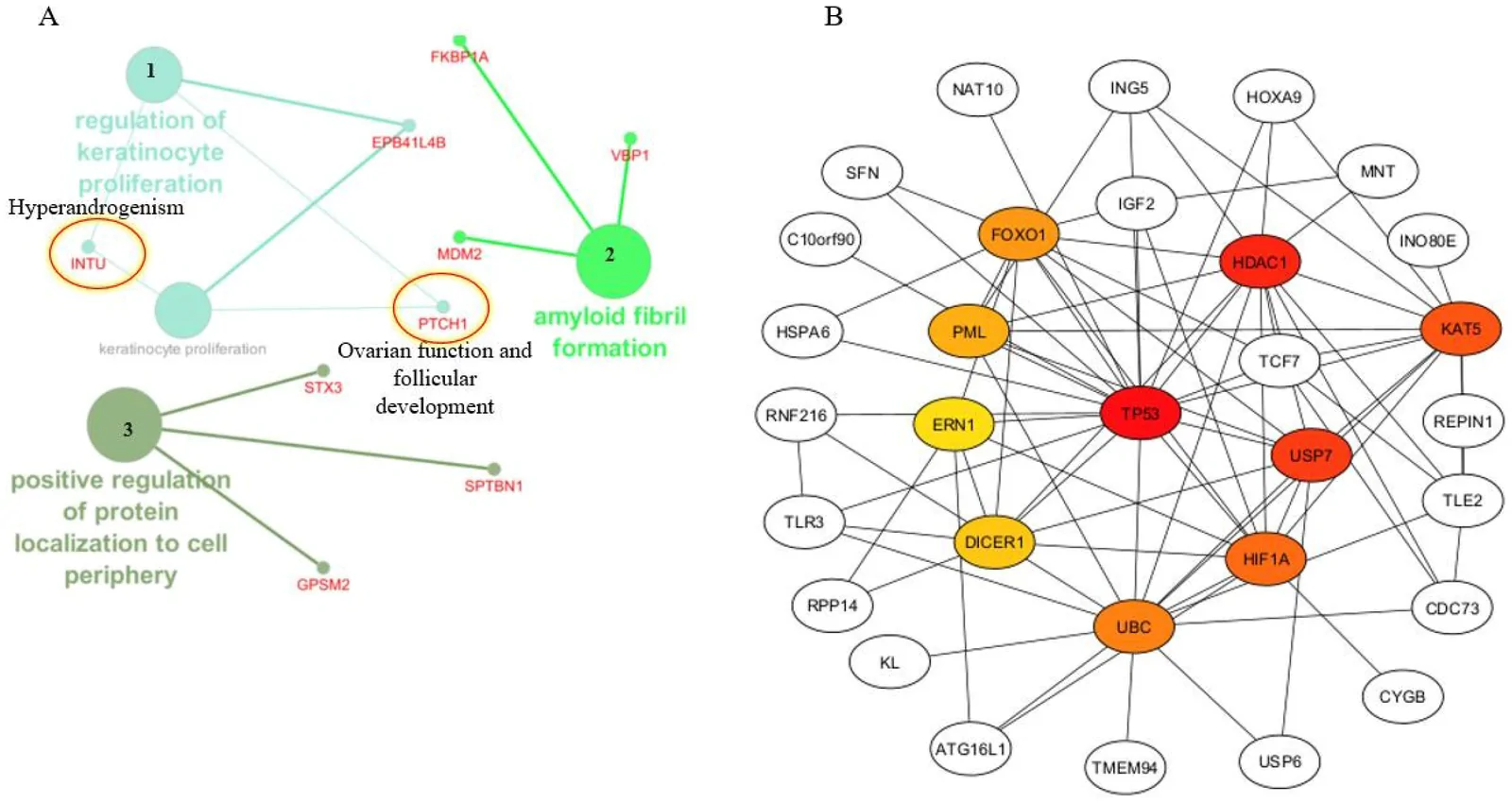
The ClueGO-generated enrichment network shows biological processes associated with hsa-miR-4508 target genes (**A**). Protein–protein interaction network built with STRING and visualized in Cytoscape. Predicted hub genes identified by the MCC algorithm in cytoHubba (**B**).

Target annotation analysis of seven candidate DE miRNAs identified potential gene targets across the samples. Among these, hsa-miR-4508-mRNA targets were consistently detected in three samples, while hsa-mir-384 and their mRNA targets were identified in only one sample. hsa-miR-4508 was selected for further analysis due to a consistent target gene identified across the sequenced samples, and this was selected for further bioinformatics analysis. The predicted target gene of hsa-miR-4508 was subjected to gene ontology and pathway enrichment analyses using Cytoscape’sv3.10.4 ClueGO (functional enrichment), and hub genes were then identified using CytoHubba (hub gene analysis). Gene ontology enrichment analysis with ClueGO shows that hsa-miR-4508 was enriched in biological processes related to keratinocyte proliferation, amyloid fibril formation, and other processes. Genes such as *INTU* and *PTCH1* were identified among the predicted clusters for hyperandrogenism and were associated with epithelial regulation and ovarian follicular development, as shown in Figure 6A. The GO enrichment analysis using ClueGO identified clusters supporting the role of hsa-miR-4508 in regulating pathways related to cellular signaling, protein modification, and ovarian dysfunction in PCOS. Furthermore, the protein–protein interactions were analyzed using CytoHubba. Hub gene analysis identified *TP53* as the top-ranked central node, followed by *HDAC1*, *KAT5*, *FOXO1*, *HIF1A*, *DICER1*, and *UBC*, shown in Figure 6B. These predicted hub genes are associated with the biological process of the cell cycle, epigenetics, insulin signaling, hypoxia, and miRNA biogenesis, all of which are crucial to PCOS pathophysiology. Target prediction analysis was performed for all seven candidate DE miRNAs using TargetScan v8.0. The top 20 predicted target mRNAs for hsa-miR-4508 and hsa-mir-384, selected for downstream analyses, are shown in Table 2. The other miRNA–mRNA targets predicted by Target Scan v8.0 are shown in Supplemental Table S3.

### 2.4. Gene Ontology Enrichment Analysis

**Figure 7:**
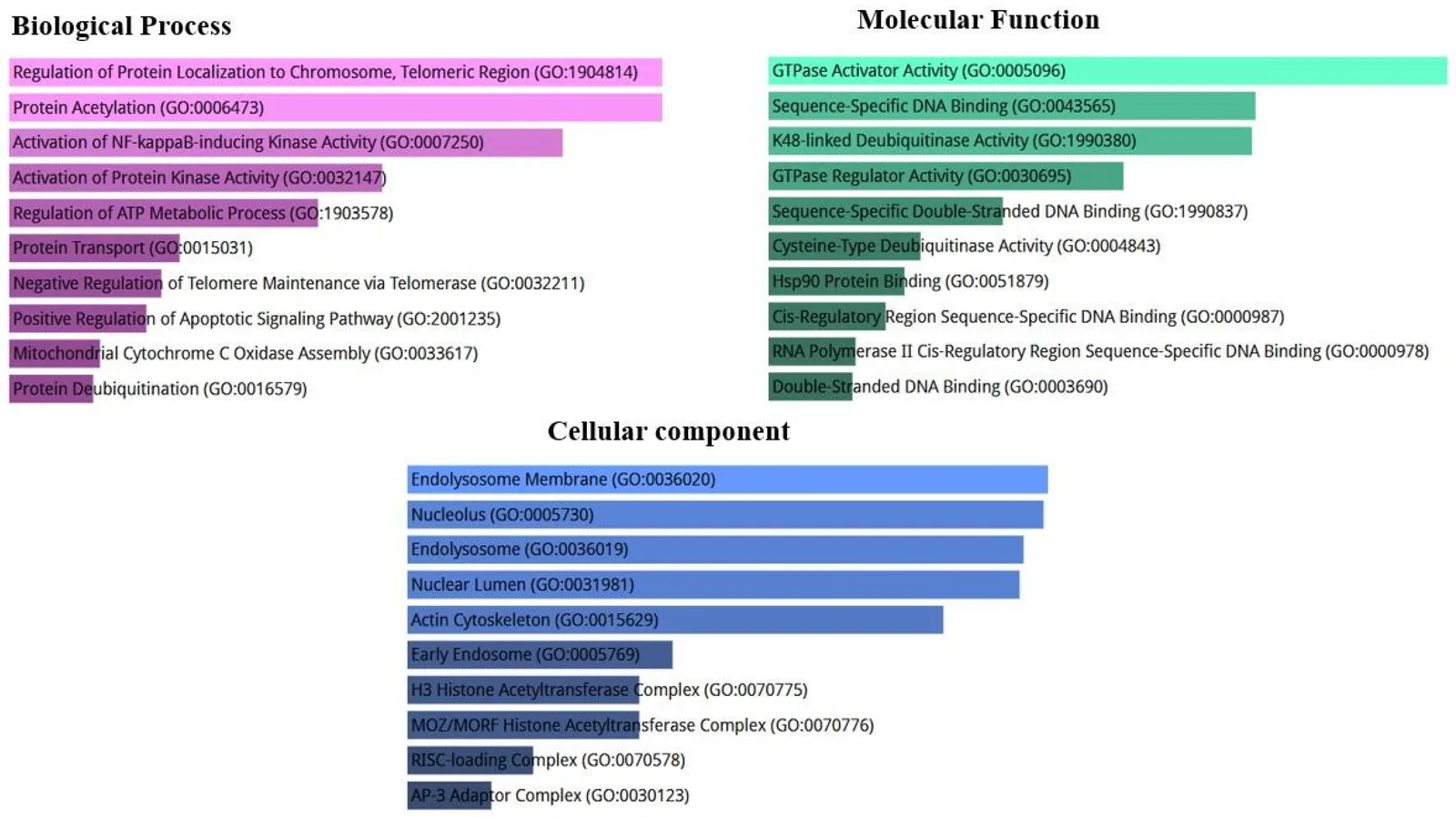
Bar plot showing significantly enriched gene ontology (GO) terms for predicted hsa-miR-4508 target genes generated using Enrichr. The enriched categories include biological processes (BPs), molecular functions (MFs), and cellular components (CCs).

Gene ontology functional analysis of the hsa-miR-4508 targets shows significant enrichment for biological terms. The biological process (BP) terms GO:2001235, positive regulation of apoptotic signaling pathway, and GO:0016579, protein deubiquitinating, govern cellular survival and metabolic maintenance, respectively, and involve protein turnover by *USP7* and *USP36*, predicting the targets of hsa-miR-4508. Molecular function (MF) terms GO:0005096 GTPase activator activity, GO:0043565 sequence-specific DNA binding, and GO:0004843 K48-linked deubiquitinase activity were identified to impact the upregulation of hsa-miR-4508 in the ubiquitin–proteasome system. Cellular component (CC) analysis identified significant enrichment in GO:0070578, RISC-loading complex, and GO:0031981, nuclear lumen, a key target of miR-4508 overexpression, potentially impairing miRNA biogenesis. Additionally, GO:0070775 and GO:0070776, which are targeted by mir-384 and predicted to regulate HDAC5, identified epigenetic remodeling in PCOS tissue. Gene ontology enrichment analysis of predicted hsa-miR-4508 target genes was performed using Enrichr by bar plots shown in Figure 7. The enrichment term, *p*-value and FDR are shown in Supplemental Table S4.

### 2.5. RT-PCR Validation of Candidate Differentially Expressed miRNAs

**Figure 8:**
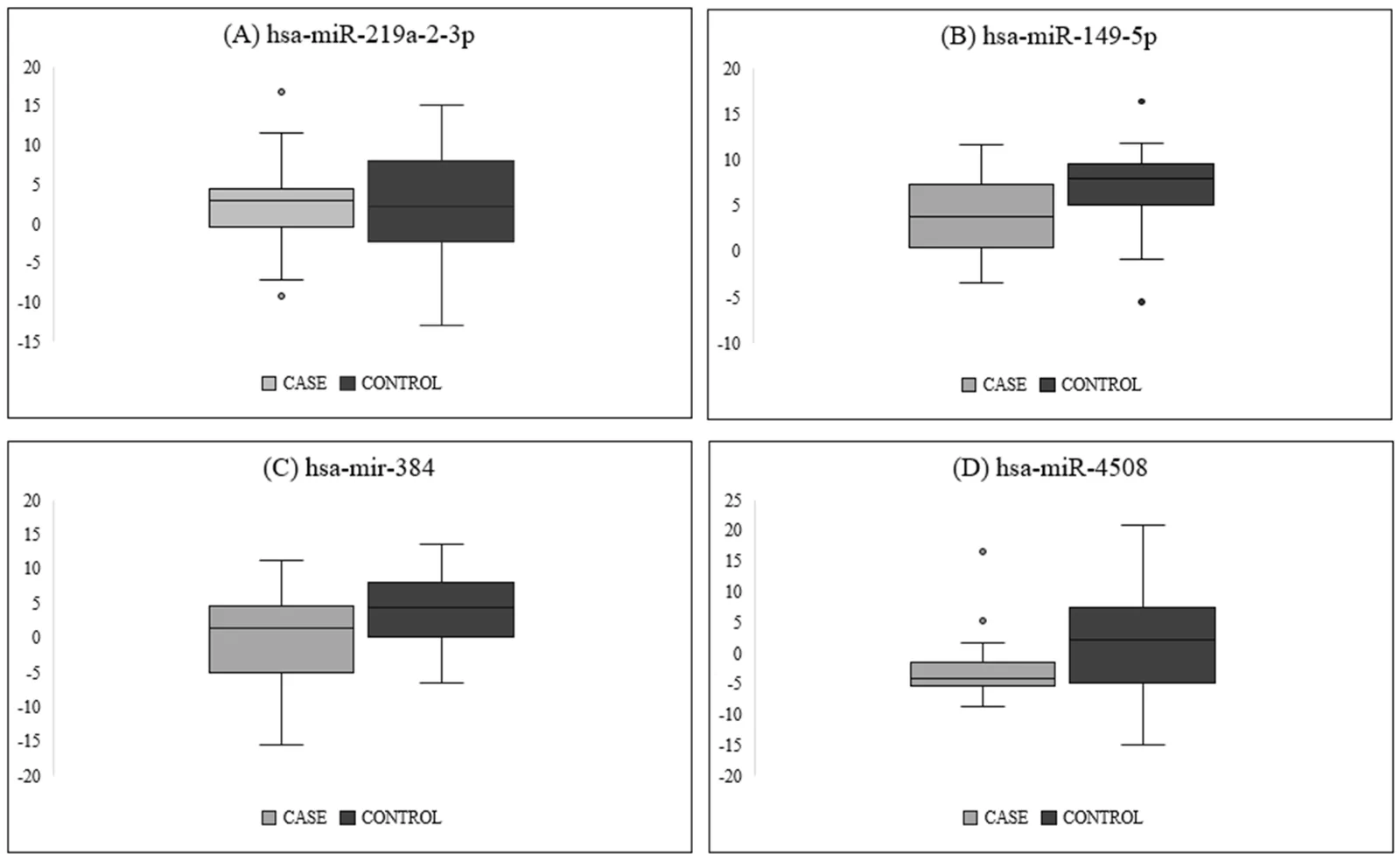
Boxplot shows the ΔCt expression values of miR-219a-2-3p, miR-149-5p, mir-384, and miR-4508 in PCOS cases and healthy controls. Significant differences in expression were found for miR-149-5p, mir-384, and miR-4508, while miR-219a-2-3p showed no difference.

Four of the candidate DE miRNAs from small RNA sequencing, such as hsa-miR-219a-2-3p, hsa-mir-384, hsa-miR-149-5p, and hsa-miR-4508, were chosen for RT-PCR validation based on the log2Fold change, base mean and their statistical analysis as mentioned in Table 1. The expression levels of miRNAs including hsa-miR-219a-2-3p, hsa-mir-384, hsa-miR-149-5p, and miR-4508 in the case and control groups are shown in Table 3. Among these miRNAs, miR-149-5p, mir-384, and miR-4508 show significant differential expression after FDR correction, with *p*-values < 0.001 and fold changes of 9.75, 16.43, and 22.13, respectively. miR-4508 showed the highest dysregulation, followed by mir-384 and miR-149-5p. Additionally, the boxplot analysis revealed distinct expression patterns for miR-149-5p, mir-384, and miR-4508, while miR-219a-2-3p showed considerable overlap, as shown in Figure 8. The four-miRNA RT-PCR gene expression ΔCT, ΔΔCt, 2^-ΔΔCt, LOG2 fold change values are reported in Supplemental Table S5.

## 3. Discussion

Polycystic ovary syndrome (PCOS) is a highly complex and diverse hormonal disorder that affects women across reproductive, metabolic, and psychological domains [15]. Current understanding of this syndrome involves a complex interaction of genetic, epigenetic, and environmental factors, yet a single unifying mechanism to explain these diverse observations has not been identified [16]. A major challenge in managing PCOS is its phenotypic heterogeneity, which varies substantially across genotypes, ethnic groups, and environmental contexts [17]. Traditional markers such as LH, FSH, and androgen levels are useful but often insufficient for early diagnosis, as they are influenced by physiological cycles and typically confirm the syndrome only after symptoms appear [18]. Furthermore, current treatments focus on managing endpoints like insulin resistance or infertility, not on underlying molecular issues [19]. Nowadays, identifying specific miRNA profiles using high-throughput sequencing is promising for investigating the pathophysiology of PCOS. Serum was chosen as the study biomaterial because it is minimally invasive and practical for large-scale screening; circulating miRNAs in serum are stable due to their association with Argonaut proteins, which protect them from degradation; and serum-derived miRNA profiling has been consistently reported across PCOS studies, enabling cross-study comparisons. Previous miRNA studies in PCOS used various biomaterials, including follicular fluid, granulosa cells, plasma exosomes, and blood. Follicular fluid miRNAs reflect intraovarian changes, whereas serum miRNAs capture systemic dysregulation and enable non-invasive testing.

In this study, we used small RNA sequencing from serum samples to identify candidate differentially expressed circulating miRNAs in Indian women with PCOS. Our exploratory sequencing results identified seven candidate miRNAs that show differential expression, suggesting potential regulatory networks underlying metabolic and ovarian dysfunction. These preliminary findings of miRNAs hsa-miR-219a-2-3p, hsa-mir-384, hsa-miR-149-5p, hsa-miR-3182, hsa-miR-3960, and hsa-miR-4508 are associated with various clinical and pathological conditions, including cancer, metabolic disorders, and autoimmune diseases. Notably, hsa-miR-219a-2-3p, hsa-mir-384, and hsa-miR-149-5p exhibit strong statistical significance, after FDR correction showing upregulation in PCOS cases.

The seven candidate miRNAs identified in this study are associated with oncological, metabolic, and inflammatory processes, suggesting a potential role in the pathophysiology of PCOS. They mainly function in regulating cell growth and apoptosis, metabolic and insulin signaling, as well as vascular and epigenetic processes. Regarding cell growth and apoptosis, hsa-miR-219a-2-3p exhibits diverse expression profiles across various disease conditions, including liver cancer, where it is significantly upregulated in serum small extracellular vesicles (sEVs) [20]. Thyroid conditions, such as benign nodules and PTC, are associated with upregulation [21]. In neurological malignancies, hsa-miR-219a-2-3p is downregulated in glioblastoma and TERTp-mutated tonsillar squamous cell carcinoma [22]. In many oncological studies, hsa-mir-384 is identified as a tumor suppressor and is often expressed at low levels in esophageal squamous cell carcinoma, non-small-cell lung cancer, and prostate cancer. [23]. Its downregulation is linked to increased cell growth and metastasis [24]. Its low expression, which affects metabolic function in obesity, indicates significant downregulation in obese males and suggests a potential role in regulating insulin signaling [25].

In metabolic and inflammatory signaling, hsa-miR-149-5p is predicted through computational analysis to affect the *MAPK* and *PI3K/Akt* signaling pathways via ERbB3 downregulation in systemic lupus erythematosus [26]. And also, it functions as either an oncogene or a tumor suppressor in hepatocellular carcinoma [27], inhibiting growth and metastasis via methylenetetrahydrofolate reductase [28]. The vascular conditions and epigenetic regulation of hsa-miR-3182 were identified as tissue markers in hepatocellular carcinoma [29], and correlate with claudication distance in patients with lower-extremity artery disease (LEAD) [30]. hsa-miR-3960 is similarly downregulated in TERTp-mutated tonsillar squamous cell carcinoma [31], is downregulated in arteriovenous fistulas and plasma exosomes in patients with diffuse large B-cell lymphoma (DLBCL) [32], and implicated in neuronal protection through the circHtra1-miR-3960-GRB10 axis in the traumatic brain injury model [33]. hsa-miR-4508 was elevated in plasma in preeclampsia, regulating genes like *SLC43A3* and *SLC12A4* for fetal development [34], has been seen in patients after receiving the sedative dexmedetomidine [35] and was downregulated in pediatric beta-thalassemia patients [36].

In the context of PCOS-related infertility, these candidate DE miRNAs have been found to inhibit a protein network involving XIAP, INSR, HIF1A, and IGF2. Their overexpression may affect granulosa cell survival and insulin signaling pathways. The increased levels of hsa-miR-4508 and mir-384 target *HIF1A* and *IGF2*, impairing ovarian response and follicular growth signaling. Regarding epigenetic remodeling, hsa-mir-384 targets *HDAC5*, which modifies histone acetyltransferase complexes, suggesting that epigenetic changes occur in PCOS tissues. HDAC-mediated epigenetic modifications regulate androgen biosynthesis genes like CYP17A1, suggesting a link between mir-384 dysregulation and PCOS hyperandrogenism. A higher level of miR-4508 targets DICER1, which regulates the miRNA feedback loop, impairing its function in biogenesis. Based on network analysis predictions, these dysregulated miRNAs may be linked to metabolic and reproductive issues in PCOS and should be validated in larger cohorts. Similarly, hsa-miR-149-5p affects the PI3K/AKT pathway, crucial for ovarian follicular development and metabolism. Dysregulation may lead to impaired follicular selection and anovulation.

This study identifies the hub genes through a computational approach—*TP53*, *FOXO1*, *HIF1A*, and *HDAC1*—which are key nodes in PCOS-related regulatory networks. TP53 regulates granulosa cell apoptosis and follicular atresia. Its dysregulation may contribute to impaired follicular selection in PCOS. *FOXO1* regulates FSH signaling and insulin sensitivity in granulosa cells, and its suppression could lead to anovulation. *HIF1A* regulates follicular hypoxia and angiogenesis in the ovary; HDAC1-related epigenetic changes may affect steroidogenesis and androgen biosynthesis. Furthermore, it requires experimental validation at the mRNA and protein levels in PCOS tissue samples.

**Figure 9:**
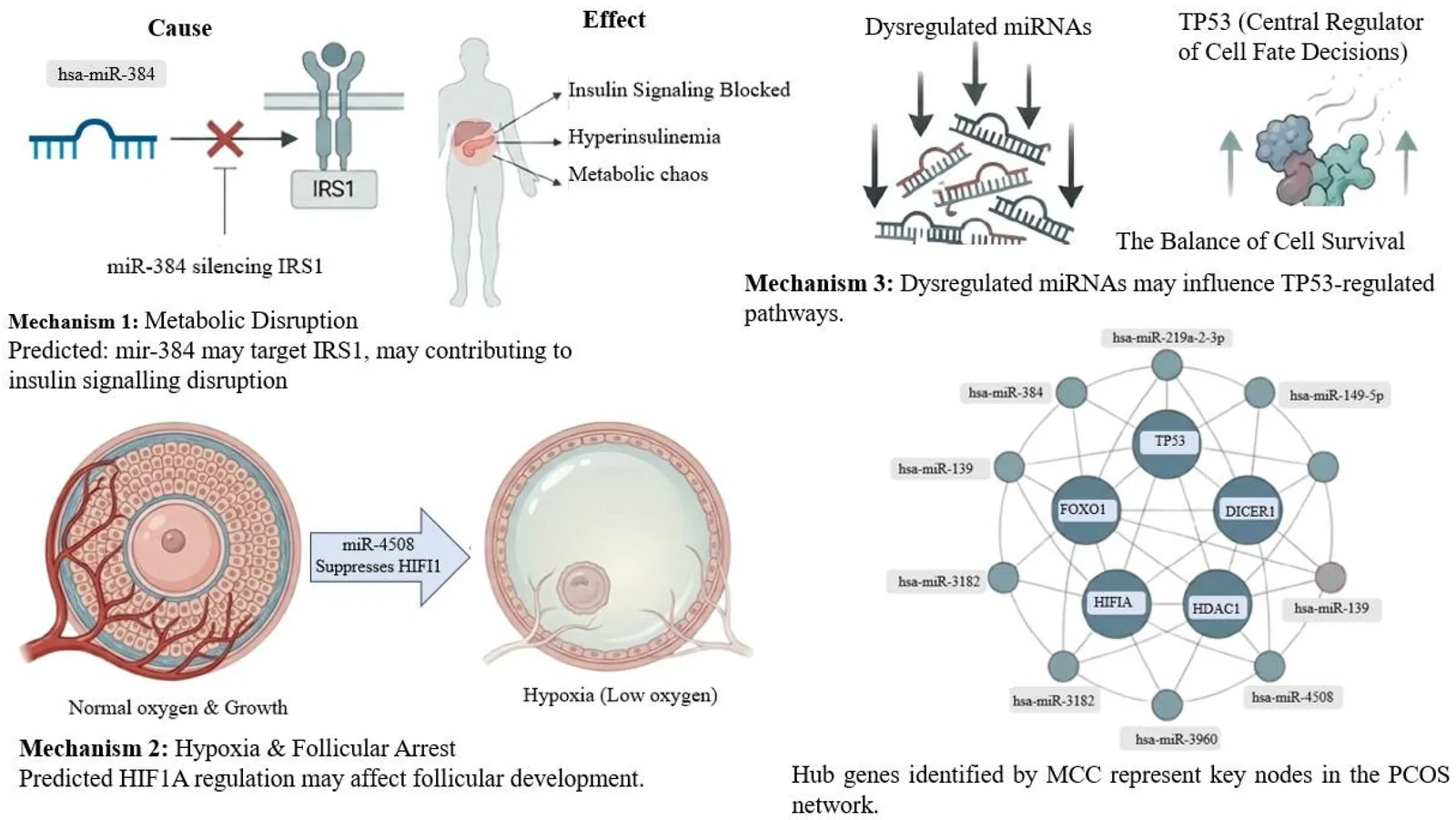
Proposed mechanistic framework showing the predicted roles of dysregulated miRNAs and hub genes in PCOS pathophysiology.

Based on the findings, a summary of PCOS pathogenesis involving hsa-miR-4508 metabolic disruption and hypoxic follicular arrest is shown in Figure 9, including *IRS1* silencing by hsa-mir-384 and dysregulated miRNAs disrupting the *TP53* regulator along with the miRNA hub gene network. The present study displayed an analysis of major miRNAs that regulate the target gene associated with PCOS. The differential expression analysis identifies three significant upregulated miRNAs: hsa-miR-219a-2-3p, hsa-mir-384, and hsa-miR-149-5p. The analysis identifies the key regulatory pathways and their biological processes influenced by these miRNAs.

The gene ontology annotation identified by the computational approach shows that the biological function of hsa-mir-384 may regulate *IRS1*, which affects insulin signaling and is involved in PCOS-related hyperinsulinemia, and disturbs metabolism in PCOS. hsa-miR-149-5p is predicted to target *AKT1* and *FasL*, likely leading to early granulosa cell apoptosis. This may explain the clinical findings of follicular arrest and the failure to select a dominant follicle. The miRNAs, such as hsa-mir-384 and hsa-miR-4508, will serve as regulators of biological pathways in PCOS. hsa-mir-384 is predicted to target *XIAP*, which inhibits cell death and is a positive regulator of apoptosis. The overexpression of this miRNA, which leads to *XIAP* silencing, is linked to follicular arrest and granulosa cell death observed in PCOS. Network analysis suggested that hsa-miR-4508 targets HIF1A, IGF2, and BMPR2, which are involved in ovarian responses to hypoxia and growth cues, which are essential for follicle development and maturation. GTPase activator activity, sequence-specific DNA binding, cysteine-type deubiquitinase activity, and K48-linked deubiquitinase activity were also identified as key molecular hubs that enhance the effect of upregulated hsa-miR-4508 on the ubiquitin–proteasome system by the gene ontology process.

Furthermore, functional enrichment supports these findings, showing the activity of protein deubiquitinating enzymes that target *USP7* and *USP36*, suggesting a disruption in protein turnover, and the RISC-loading complex DICER1. The regulatory hub genes were identified as key metabolic and growth factors using ClueGO and CytoHubba. Based on network analysis, these genes are regulators of the cell cycle, epigenetics, insulin signaling, hypoxia adaptation, and miRNA production.

The present study also analyses RT-PCR validation, which shows the dysregulation of miR-149-5p, mir-384, and miR-4508 in PCOS patients compared with healthy controls, whereas miR-219a-2-3p shows no significant changes. The upregulation of miR-149-5p, mir-384, and miR-4508 suggests that they may contribute to the molecular mechanisms of PCOS. MicroRNAs regulate gene expression post-transcriptionally and are crucial for ovarian function, steroid hormone production, insulin signaling, inflammation, and follicle development, all of which are essential processes associated with PCOS. Among the validated miRNAs, miR-4508 showed the highest fold change, followed by mir-384 and miR-149-5p, suggesting these miRNAs may play significant roles in disease-related regulatory networks. The RT-PCR results after FDR correction show these circulating candidate miRNAs for PCOS diagnosis, along with their associated endocrine and metabolic complications. Overall, these findings identify miR-149-5p, mir-384, and miR-4508 as preliminary candidate differentially expressed miRNAs that require validation in larger cohort samples and also in further functional validation studies.

## 4. Materials and Methods

### 4.1. Sample Selection

This study was conducted as a preliminary, exploratory pilot to evaluate the methodology and to provide guidance for future confirmatory research. Small sample sizes are commonly used in the discovery phase of high-throughput miRNA sequencing research, including published PCOS miRNA sequencing studies that start with small initial cohorts before moving to larger validation stages. This pilot study aims to identify candidate miRNAs through miRNA sequencing and to validate them in RT-PCR in PCOS cases and age-matched controls. The four samples chosen for next-generation sequencing were not selected at random. Before processing samples for small RNA library preparation and sequencing, RT-PCR gene expression analysis for (miRNA-21, miRNA-146a) was performed on 50 PCOS cases and 50 age-matched controls. These miRNAs were chosen based on published literature.

This pilot study was initiated primarily due to the lack of existing small RNA sequencing data on circulating miRNAs in Indian PCOS populations. Because miRNA expression varies by ethnicity, diet, lifestyle, and genetics, making assumptions based on other populations is unreliable. As a result, a literature-based power calculation was not appropriate for this group. This exploratory transcriptomics study focuses on identifying candidate miRNAs using small RNA sequencing.

### 4.2. Study Participant and Sample Collection

PCOS cases (*n* = 3) and age-matched controls (*n* = 1) were recruited from patients attending the gynecology OPD at Aarupadai Veedu Medical College and Hospital. The sample selection for both pre-screening and sequencing phases was based on predefined clinical criteria. Based on the Rotterdam criteria for diagnosis, at least two of the following were required for recruitment in the study: (1) oligo- and/or anovulation, (2) clinical and hyperandrogenism, and (3) polycystic ovaries identified by ultrasonography.

The inclusion criteria for this study were participants aged 18–35 years who either met the Rotterdam criteria for PCOS (cases) or other endocrine disorders. Controls were matched by age. Exclusion criteria included the presence of known thyroid disorders, Cushing’s syndrome, or any other endocrine conditions. Additionally, participants using oral contraceptive pills, which can affect circulating miRNA levels, were excluded from the study. After obtaining informed consent from the study participants, 3 mL of blood was collected and sent for miRNA processing.

### 4.3. miRNA Extraction

Serum samples were collected in serum collection tubes from patients; the serum was then separated by centrifugation at 8000× *g* for 2 min and stored at −80 °C. The miRNA was extracted using a QIAamp miRNAeasy serum/plasma kit (Qiagen, Germantown, MD, USA). The RNA samples were quantified using Qubit RNA BR Assay. RNA purity was checked using QIAxpert, and RNA integrity was assessed on Tape Station using RNA Screen Tapes.

### 4.4. miRNA Library Preparation and Sequencing

The miRNA libraries were prepared using the QIAseq miRNA Library Kit protocol (QIAGEN, Cat# 331565, Hilden, Germany). Adapters are ligated sequentially to the 3′ and 5′ ends of miRNAs. The ligated miRNAs are then converted to cDNA using a reverse transcription primer containing UMIs (Unique Molecular Identifiers) to minimize sequence-dependent ligation and capture of canonical miRNAs and isomiRs. After cDNA cleanup, libraries were amplified with 95 °C for 15 min, 22 cycles of 95 °C for 15 s, 60 °C for 30 s, 72 °C for 15 s, and then 72 °C for a 2 min final extension. After library amplification, the miRNA libraries were cleaned up using a streamlined magnetic bead-based method. The libraries were checked for fragment size distribution on a Tape Station using D1000 DNA ScreenTapes (Agilent, Cat# 5067-5582, Santa Clara, CA, USA). Prepared libraries were quantified using the Qubit High-Sensitivity Assay. The libraries were then pooled and loaded onto the Illumina platform (NextSeq 550) using paired-end sequencing to a minimal depth. In addition, hemolysis was evaluated in the sequencing read counts of erythrocyte-enriched miRNA hsa-miR-451a and hemolysis-insensitive miRNA hsa-miR-23a in the known miRNA count matrix. The distribution of canonical miRNAs and their corresponding isomiRs was identified as part of the sequencing quality assessment.

### 4.5. Identification of miRNAs by Differential Expression

The fast QC tool was used to assess read preprocessing, including the base quality score distribution, average base content per cycle, and GC content, to retain high-quality reads for further analysis. Adapter sequence and low-quality bases were removed by using Cutadapt v.1.15. The trimmed reads with lengths between 17 and 30 bp were filtered, and then the reads were aligned to the miRbase database [37] to identify miRNA reference sequences. Raw read counts were normalized using DESeq2 for differential expression analysis. Candidate differentially expressed miRNAs (DEmiRNAs) between the PCOS and control groups were identified using DESeq2 [38]. Thresholds for significance were a minimum fold change in Log2FC > 1of *p*-value < 0.05.

### 4.6. Target Prediction and Network Interaction

The target predictions for the miRNAs were performed by using Target Scan v8.0 DB, which provides miRNA-target details through various validated tools and prediction approaches [39]. It allows us to confirm miRNA targets across 3′-UTR, 5′-UTR, and CDS regions of all miRNAs. Based on the miRNA-mRNA target, a PPI network was constructed by using the String tool (European Molecular Biology Laboratory, Heidelberg, Germany) [40]. Furthermore, key nodes within the PPI network were identified as hub genes using the CytoHubba plugin in Cytoscape [41]. Hub genes were selected based on the Maximal Clique Centrality (MCC) algorithm for the biological network [42].

### 4.7. Gene Ontology (GO) and Functional Enrichment

Functional annotation of the identified target miRNAs was assessed by using gene ontology (GO) functional analysis by the Ensembl BioMart database [43]. GO analysis classified gene functions into: biological processes (BPs), cellular components (CCs), and molecular functions (MFs). A statistical threshold of *p* < 0.05 was used to identify enriched GO terms and pathways. The pathway enrichment analysis for the target miRNAs was performed by using Enricher [44].

### 4.8. Gene Expression Analysis by RT-PCR

As mentioned in Section 4.3, miRNA was extracted and converted into cDNA using the TaqMan™ Advanced miRNA cDNA Synthesis Kit (Thermo Fisher Scientific, Waltham, MA, USA) according to the kit’s protocol. PCR amplification for gene expression of target miRNAs was carried out using RT-PCR (Applied Biosystems, Foster City, CA, USA). The total reaction volume was 10 µL, which included 5 µL of SYBR Green master mix, 1 µL of forward primer, 1 µL of reverse primer, 1 µL of cDNA, and 2 µL of nuclease-free water. The optimal thermocycling conditions followed 95 °C for 15 min, 94 °C for 15 s, 55 °C for 30 s, and 72 °C for 30 s for 40 cycles. The primers used for the study are reported in Supplemental Table S6. RNU6 was used as the internal control, and each target miRNA was normalized to calculate ∆Ct. The fold change of miRNAs in PCOS with respect to control was calculated using 2-∆∆CT. The sample size was calculated by using a two-sample mean comparison with a 5% significance level and 90% power. Based on the sample size calculation, 47 participants were required for both groups. To improve study robustness, (*n* = 50) cases and (*n* = 50) controls were recruited for the study.

### 4.9. Statistical Analysis

Statistical analyses were performed using SPSSv29.0. All continuous variables were expressed as mean ± standard deviation (SD). miRNA expression was analyzed using Student’s *t*-test, and a *p*-value < 0.01 was considered significant. *p*-values were adjusted for multiple testing using the Benjamini–Hochberg false discovery rate (FDR).

## 5. Conclusions

This exploratory study provides a preliminary analysis of circulating serum microRNAs in Indian women with polycystic ovary syndrome (PCOS) using small RNA sequencing. A total of seven candidate DE miRNAs were identified, of which six miRNAs were upregulated, and one miRNA was downregulated. Integrated bioinformatics and network analysis showed that these miRNAs play roles in key biological processes related to PCOS pathophysiology, including disruption of insulin signaling, granulosa cell apoptosis, hypoxia response, ubiquitin-mediated protein degradation, and epigenetic remodeling. Hub gene identification highlighted TP53, FOXO1, HIF1A, HDAC1, and DICER1 as key regulators, indicating disrupted metabolic and reproductive pathways.

The RT-PCR validation of four miRNAs, miR-149-5p, mir-384, miR-4508, and hsa-miR-219a-2-3p, with fold changes of 9.75, 16.43, 22.13, and 1.05, was analyzed for 50 PCOS patients and 50 age-matched controls. However, these findings are preliminary, and these candidate miRNAs require larger cohort samples with clinical, hormonal, metabolic, and phenotypic profiling to validate these findings and determine the clinical relevance of the identified candidate miRNAs in PCOS for the biomarker studies.

### Limitation of the Study

Although the sample size was limited, all findings should be considered preliminary and exploratory. The study used four samples for small RNA sequencing, which was the primary limitation, reducing the statistical robustness of the DESeq2 differential expression analysis. Future studies involving validation in larger independent cohorts are needed to confirm the diagnostic and therapeutic relevance of these miRNAs and to advance precision medicine approaches in PCOS management. This is, to our knowledge, the first pilot miRNA exploratory study in an Indian PCOS cohort. This study reports a preliminary serum miRNA signature in an Indian cohort and suggests that these circulating miRNAs may serve as preliminary candidate miRNAs for PCOS.

## Figures and Tables

**Table 1:** Differential expression analysis of candidate miRNAs was performed using small RNA sequencing. Expression levels were normalized with DESeq2, and significance was determined based on log2 fold change (log2FC) and adjusted *p*-value (Padj). Upregulation in PCOS is represented by a positive log2FC, while downregulation is indicated by a negative log2FC.

S.NO	miRNA	Base Mean	log2Fold Change	lfcSE	Adjusted *p*-Value	*p*-Value
1.	hsa-miR-219a-2-3p	148.5081854	21.2558763	4.152448	0.000297	3.07358
2.	hsa-mir-384	71.21918429	20.23806926	4.091728	0.000366	7.57175
3.	hsa-miR-149-5p	28.32581194	18.95819574	4.040066	0.00087	2.69825
4.	hsa-miR-3182	47.40053416	7.773959564	3.387848	0.999725	0.02175
5.	hsa-miR-3960	32.12972018	7.213063031	3.34857	0.999725	0.03123
6.	hsa-miR-4508	40.09017268	7.532004221	3.831972	0.999725	0.04934
7.	hsa-mir-139	6.66487296	−7.415739486	3.3251	0.999725	0.02573

**Table 2:** The top 20 predicted mRNA targets of hsa-miR-4508 and hsa-mir-384 from TargetScan v8.0. Ensembl transcript IDs (ENST) and gene symbols are provided. MiRNA-mRNA targets were selected based on prediction scores and sample consistency.

miR-4508			mir-384	
S.NO	Ensembl ID	mRNA	Ensembl ID	mRNA
1	ENST00000491381.1	SNX21	ENST00000390677.2	TAS2R13
2	ENST00000367435.3	CDC73	ENST00000372299.3	KLF17
3	ENST00000542802.3	USP36	ENST00000329959.4	WBSCR16
4	ENST00000426690.2	KL	ENST00000240055.3	NFYB
5	ENST00000361573.2	SLC9A8	ENST00000388822.5	METTL14
6	ENST00000269919.6	PGPEP1	ENST00000361905.4	TEAD1
7	ENST00000408895.2	C3orf36	ENST00000434739.3	GOLGA6D
8	ENST00000383075.3	ZIC4	ENST00000344700.3	RPS10
9	ENST00000563197.1	INO80E	ENST00000318596.7	SLC25A42
10	ENST00000250066.6	USP6	ENST00000371591.1	ZFYVE9
11	ENST00000398322.3	ACBD4	ENST00000205402.5	DLD
12	ENST00000267328.3	RAB20	ENST00000406462.2	GPSM2
13	ENST00000375678.3	C20orf112	ENST00000376088.3	CLCN5
14	ENST00000589342.1	CYGB	ENST00000312240.2	OR5M3
15	ENST00000507848.1	FAM153C	ENST00000284601.3	PPP1R3A
16	ENST00000529859.1	PCDHA5	ENST00000330997.4	ZNF285
17	ENST00000343216.3	CXXC11	ENST00000370251.3	ZNF275
18	ENST00000222990.3	SNX8	ENST00000482457.2	ZNF80
19	ENST00000332438.4	CCR10	ENST00000545173.2	EIF3K
20	ENST00000174618.4	MNT	ENST00000545173.2	EIF3K

**Table 3:** Differential expression of the candidate miRNAs between case and control samples. Fold change and log2FC were calculated from a group of ΔCt.

miRNA	Mean ΔCt ± SD (Case)	Mean ΔCt ± SD (Control)	Fold Change	Log2FC	Adjusted *p*-Value	*p*-Value
miR-219a-2-3p	2.22 ± 4.78	2.29 ± 6.00	1.05	0.07	0.949	0.949406
miR-149-5p	4.03 ± 3.84	7.31 ± 3.76	9.75	3.28	1.1 × 10^−4^ *	3.08 × 10^−5^
mir-384	0.01 ± 6.62	4.05 ± 4.51	16.43	4.04	9.4 × 10^−4^ *	5.00 × 10^−4^
miR-4508	−3.00 ± 4.14	1.47 ± 7.48	22.13	4.47	9.4 × 10^−4^ *	3.90 × 10^−4^

* Indicates statistical significance after false discovery rate (FDR) adjustment (*p* < 0.05).

## Data Availability

The raw small RNA sequencing data generated during this study have been deposited in the National Center for Biotechnology Information (NCBI) Sequence Read Archive (SRA) under BioProject accession PRJNA1483023. The associated BioSample accessions are SAMN61126027, SAMN61126028, SAMN61126029, and SAMN61126030. As recommended by NCBI, the Bio Project accession PRJNA1483023 should be used to access and cite the sequencing data associated with this study.

## Supplementary Materials

The following supporting information can be downloaded at https://www.mdpi.com/article/10.3390/ncrna12040030/s1. Supplemental Table S1. Sequence quality control information and isomiRs identified from small RNA sequencing. Supplemental Table S2. Differential expression analysis of miRNAs identified from small RNA sequencing. Supplemental Table S3. Predicted miRNA–mRNA target interactions for the miRNAs with Ensembl gene IDs. Supplemental Table S4. Gene Ontology (Biological Process, Molecular Function, and Cellular Component) enrich-ment analysis with *p* values and adjusted *p* values. Supplemental Table S5. RT-PCR gene expression values for 50 PCOS cases and 50 healthy controls. Supplemental Table S6. Primer sequences used for the RT-PCR gene expression study.

## Abbreviation

PCOS	Polycystic Ovary Syndrome
DE miRNAs	Differentially Expressed miRNAs
UMIs	Unique Molecular Identifiers
miRNAs	MicroRNAs
RT-PCR	Real-Time-Polymerase Chain Reaction
hsa	Homo sapiens
MCC	Maximal Clique Centrality
GO	Gene Ontology
BP	Biological Processes
CC	Cellular Components
MF	Molecular Functions

## References

[1] Teede H.J., Misso M.L., Costello M.F., Dokras A., Laven J., Moran L., Piltonen T., Norman R.J., International P.N. (2018). Recommendations from the international evidence-based guideline for the assessment and management of polycystic ovary syndrome. Hum. Reprod..

[2] Krentowska A., Ponikwicka-Tyszko D., Łebkowska A., Adamska A., Sztachelska M., Milewska G., Hryniewicka J., Wołczyński S., Kowalska I. (2024). Serum expression levels of selected microRNAs and their association with glucose metabolism in young women with polycystic ovary syndrome. Pol. Arch. Intern. Med..

[3] Bozdag G., Mumusoglu S., Zengin D., Karabulut E., Yildiz B.O. (2016). The prevalence and phenotypic features of polycystic ovary syndrome: A systematic review and meta-analysis. Hum. Reprod..

[4] Cirillo F., Catellani C., Lazzeroni P., Sartori C., Nicoli A., Amarri S., La Sala G.B., Street M.E. (2019). MiRNAs Regulating Insulin Sensitivity Are Dysregulated in Polycystic Ovary Syndrome (PCOS) Ovaries and Are Associated with Markers of Inflammation and Insulin Sensitivity. Front. Endocrinol..

[5] Zhang F., Li S., Zhang T., Yu B., Zhang J., Ding H.G., Ye F.J., Yuan H., Ma Y.Y., Pan H.T. (2021). High throughput microRNAs sequencing profile of serum exosomes in women with and without polycystic ovarian syndrome. PeerJ.

[6] Erson A.E., Petty E.M. (2008). MicroRNAs in development and disease. Clin. Genet..

[7] Kyriazi A.A., Papiris E., Kitsos Kalyvianakis K., Sakellaris G., Baritaki S. (2020). Dual Effects of Non-Coding RNAs (ncRNAs) in Cancer Stem Cell Biology. Int. J. Mol. Sci..

[8] Bartel D.P. (2004). MicroRNAs: Genomics, biogenesis, mechanism, and function. Cell.

[9] Arancio W., Amato M.C., Magliozzo M., Pizzolanti G., Vesco R., Giordano C. (2018). Serum miRNAs in women affected by hyperandrogenic polycystic ovary syndrome: The potential role of miR-155 as a biomarker for monitoring the estroprogestinic treatment. Gynecol. Endocrinol..

[10] Santonocito M., Vento M., Guglielmino M.R., Battaglia R., Wahlgren J., Ragusa M., Barbagallo D., Borzì P., Rizzari S., Maugeri M. (2014). Molecular characterization of exosomes and their microRNA cargo in human follicular fluid: Bioinformatic analysis reveals that exosomal microRNAs control pathways involved in follicular maturation. Fertil. Steril..

[11] Moreno-Moya J.M., Vilella F., Simon C. (2014). MicroRNA: Key gene expression regulators. Fertil. Steril..

[12] Shen K.Y., Dai X.L., Li S., Huang F., Chen L.Q., Luo P., Qu X.L. (2023). Specific expression profile of follicular fluid-derived exosomal microRNAs in patients with diminished ovarian reserve. BMC Med. Genom..

[13] Potla P., Ali S.A., Kapoor M. (2021). A bioinformatics approach to microRNA-sequencing analysis. Osteoarthr. Cartil. Open.

[14] Devarbhavi P., Telang L., Vastrad B., Tengli A., Vastrad C., Kotturshetti I. (2021). Identification of key pathways and genes in polycystic ovary syndrome via integrated bioinformatics analysis and prediction of small therapeutic molecules. Reprod. Biol. Endocrinol..

[15] Ebrahimi S.O., Reiisi S., Parchami Barjui S. (2018). Increased risk of polycystic ovary syndrome (PCOS) associated with CC genotype of miR-146a gene variation. Gynecol. Endocrinol..

[16] Prabhu B.N., Kanchamreddy S.H., Sharma A.R., Bhat S.K., Bhat P.V., Kabekkodu S.P., Satyamoorthy K., Rai P.S. (2021). Conceptualization of functional single nucleotide polymorphisms of polycystic ovarian syndrome genes: An in silico approach. J. Endocrinol. Investig..

[17] Teede H., Deeks A., Moran L. (2010). Polycystic ovary syndrome: A complex condition with psychological, reproductive and metabolic manifestations that impacts on health across the lifespan. BMC Med..

[18] Liu Y., Shi X., Xu B., Wang Z., Chen Y., Deng M. (2023). Differential expression of plasma derived exosomal miRNAs in polycystic ovary syndrome as a circulating biomarker. Biomed. Rep..

[19] Krishna M.B., Johnson B.S., Vasudevan M., Pillai S.M., Laloraya M. (2023). miRNA-mRNA Network in PBMCs of PCOS Women Identifies Overactivated Stress-Activated Kinases. Cell. Physiol. Biochem..

[20] Fan T., Zhou B., Chen H., Liu L.P., Ni Y., Zhang W., Ye L., Chen Y., Zhang D., Yang S. (2026). Novel serum small extracellular vesicle miRNAs with multi-target RCA-CRISPR sensor for liver cancer detection. J. Transl. Med..

[21] Cui W., Xuan T., Liao T., Wang Y. (2024). From sequencing to validation: NGS-based exploration of plasma miRNA in papillary thyroid carcinoma. Front. Oncol..

[22] Long N., Xu X., Lin H., Lv Y., Zou S., Cao H., Chen X., Zhao Y., Qi X., Yang H. (2022). Circular RNA circPOSTN promotes neovascularization by regulating miR-219a-2-3p/STC1 axis and stimulating the secretion of VEGFA in glioblastoma. Cell Death Discov..

[23] Yu H.X., Wang X., Zhang L.N., Zhang J., Zhao W. (2019). MicroRNA-384 inhibits the progression of esophageal squamous cell carcinoma through blockade of the LIMK1/cofilin signaling pathway by binding to LIMK1. Biomed. Pharmacother..

[24] Hong Z., Fu W., Wang Q., Zeng Y., Qi L. (2019). MicroRNA-384 is lowly expressed in human prostate cancer cells and has anti-tumor functions by acting on HOXB7. Biomed. Pharmacother..

[25] Kargun K., Aygen E., Ebiloglu M.F., Alayunt N.O., Dalkilic L.K. (2025). Evaluation of miRNA Profile and Its Relationship with Metabolic Disorders in Obese and Pre-Obese Patients. Curr. Issues Mol. Biol..

[26] Ghods F., Sarikaya A., Arda N., Hamuryudan V. (2018). hsa-miR-149-5p diminish MAPK and PI3K/Akt signalling pathways through down-regulation of ERbB3 in SLE patients. Mol. Biol..

[27] Ren F.J., Yao Y., Cai X.Y., Cai Y.T., Su Q., Fang G.Y. (2021). MiR-149-5p: An Important miRNA Regulated by Competing Endogenous RNAs in Diverse Human Cancers. Front. Oncol..

[28] Li C., Li X., Wang H., Guo X., Xue J., Wang X., Ni J. (2022). MicroRNA-22-3p and MicroRNA-149-5p Inhibit Human Hepatocellular Carcinoma Cell Growth and Metastasis Properties by Regulating Methylenetetrahydrofolate Reductase. Curr. Issues Mol. Biol..

[29] El-Shqnqery H.E., Mohamed R.H., Samir O., Ayoub I., El-Sayed W.M., Sayed A.A. (2023). miRNome of Child A hepatocellular carcinoma in Egyptian patients. Front. Oncol..

[30] Zalewski D.P., Ruszel K., Stępniewski A., Gałkowski D., Feldo M., Kocki J., Bogucka-Kocka A. (2022). Relationships between indicators of lower extremity artery disease and miRNA expression in peripheral blood mononuclear cells. J. Clin. Med..

[31] Kwon M.J., Park H.Y., Lee J.S., Kim E.S., Kim N.Y., Nam E.S., Cho S.J., Kang H.S. (2023). Dysregulated microRNA Expression Relevant to TERT Promoter Mutations in Tonsil Cancer—A Pilot Study. Life.

[32] Lv L., Huang W., Zhang J., Shi Y., Zhang L. (2016). Altered microRNA expression in stenoses of native arteriovenous fistulas in hemodialysis patients. J. Vasc. Surg..

[33] Zheng P., Shu L., Ren D., Kuang Z., Zhang Y., Wan J. (2022). circHtra1/miR-3960/GRB10 Axis Promotes Neuronal Loss and Immune Deficiency in Traumatic Brain Injury. Oxid. Med. Cell. Longev..

[34] Brancaccio M., Giachino C., Iazzetta A.M., Cordone A., De Marino E., Affinito O., Vivo M., Calabrò V., Pollice A., Angrisano T. (2022). Integrated bioinformatics analysis reveals novel miRNA as biomarkers associated with preeclampsia. Genes.

[35] Yang X., Chen H., Chen Y., Birnbaum Y., Liang R., Ye Y., Qian J. (2018). Circulating miRNA Expression Profiling and Target Prediction in Patients Receiving Dexmedetomidine. Cell. Physiol. Biochem..

[36] Wang H., Chen M., Xu S., Pan Y., Zhang Y., Huang H., Xu L. (2021). Abnormal regulation of microRNAs and related genes in pediatric beta-thalassemia. J. Clin. Lab. Anal..

[37] Kozomara A., Birgaoanu M., Griffiths-Jones S. (2019). miRBase: From microRNA sequences to function. Nucleic Acids Res..

[38] Love M., Anders S., Huber W. (2014). Differential analysis of count data–the DESeq2 package. Genome Biol..

[39] Agarwal V., Bell G.W., Nam J.W., Bartel D.P. (2015). Predicting effective microRNA target sites in mammalian mRNAs. eLife.

[40] Szklarczyk D., Franceschini A., Wyder S., Forslund K., Heller D., Huerta-Cepas J., Simonovic M., Roth A., Santos A., Tsafou K.P. (2015). STRING v10: Protein-protein interaction networks, integrated over the tree of life. Nucleic Acids Res..

[41] Shannon P., Markiel A., Ozier O., Baliga N.S., Wang J.T., Ramage D., Amin N., Schwikowski B., Ideker T. (2003). Cytoscape: A software environment for integrated models of biomolecular interaction networks. Genome Res..

[42] Chin C.H., Chen S.H., Wu H.H., Ho C.W., Ko M.T., Lin C.Y. (2014). cytoHubba: Identifying hub objects and sub-networks from complex interactome. BMC Syst. Biol..

[43] Cunningham F., Allen J.E., Allen J., Alvarez-Jarreta J., Amode M.R., Armean I.M., Austine-Orimoloye O., Azov A.G., Barnes I., Bennett R. (2022). Ensembl 2022. Nucleic Acids Res..

[44] Chen E.Y., Tan C.M., Kou Y., Duan Q., Wang Z., Meirelles G.V., Clark N.R., Ma’ayan A. (2013). Enrichr: Interactive and collaborative HTML5 gene list enrichment analysis tool. BMC Bioinform..

